# Triage using a self-assessment questionnaire to detect potentially life-threatening emergencies in gynecology

**DOI:** 10.1186/1749-7922-9-46

**Published:** 2014-08-13

**Authors:** Cyrille Huchon, Alexandre Dumont, Anne Chantry, Bruno Falissard, Arnaud Fauconnier

**Affiliations:** 1 Service de gynécologie & obstétrique, CHI Poissy-St-Germain, 10 rue du champ Gaillard, BP 3082 78303, Poissy CEDEX, France; 2 Equipe d’accueil EA 7285 « Risques, cliniques et sécurité en santé des femmes et en santé périnatale », Université Versailles-Saint-Quentin en Yvelines, 78000 Versailles, France; 3 UMR 216, IRD Paris Descartes, 4 Avenue de l’Observatoire, Paris - Université, 75 006 Paris Descartes, France; 4 INSERM, UMR S953, Epidemiological Research Unit on Perinatal Health and Women’s and Children’s Health, Hôpital Cochin, Paris, France; 5 INSERM UMR S669, Université Paris Sud, Paris, France

**Keywords:** Gynecologic emergencies, Triage, Sensitivity, Questionnaire

## Abstract

**Objective:**

Acute pelvic pain is a common reason for emergency room visits that can indicate a potentially life-threatening emergency (PLTE). Our objective here was to develop a triage process for PLTE based on a self-assessment questionnaire for gynecologic emergencies (SAQ-GE) in patients experiencing acute pelvic pain.

**Methods:**

In this multicenter prospective observational study, all gynecological emergency room patients seen for acute pelvic pain between September 2006 and April 2008 completed the SAQ-GE after receiving appropriate analgesics. Diagnostic procedures were ordered without knowledge of questionnaire replies. Laparoscopy was the reference standard for diagnosing PLTE; other diagnoses were based on algorithms. In two-thirds of the population, SAQ-GE items significantly associated with PLTEs (*P* < 0.05) by univariate analysis were used to develop a decision tree by recursive partitioning; the remaining third served for validation.

**Results:**

Of 344 derivation-set patients and 172 validation-set patients, 96 and 49 had PLTEs, respectively. Items significantly associated with PLTEs were vomiting, sudden onset of pain, and pain to palpation. Sensitivity of the decision tree based on these three features was 87.5% (95% confidence interval (95% CI), 81%-94%) in the derivation set and 83.7% in the validation set. Derivation of the decision tree provided probabilities of PLTE of 13% (95% CI, 6%-19%) in the low-risk group, 27% (95% CI, 20%-33%) in the intermediate-risk group and 62% (95% CI, 48%-76%) in the high-risk group, ruling out PLTE with a specificity of 92.3%; (95% CI, 89%-96%). In the validation dataset, PLTE probabilities were 16.3% in the low-risk group, 30.6% in the intermediate-risk group, and 44% in the high-risk group, ruling out the diagnosis of PLTE with a specificity of 88.6%.

**Conclusion:**

A simple triage model based on a standardized questionnaire may assist in the early identification of patients with PLTEs among patients seen in the gynecology emergency room for acute pelvic pain.

## Introduction

Acute pelvic pain is the leading reason for gynecological emergency room visits [1]. However, only a minority of these patients require emergency surgery. Thus, in a study of 205 patients seen at the gynecological emergency room of a French hospital in 2011, only 24 (12%) required hospital admission and 9 (4.5%) surgical treatment [2]. The early identification of patients with potentially life-threatening emergencies (PLTEs) requiring prompt surgical treatment is crucial [3].

In general emergency rooms, nurses typically prioritize patients to ensure that those with serious conditions are seen first by the emergency physicians. Triage scales such as the Emergency Severity Index [4] are used to determine whether medical care is required immediately, within a few minutes, within the next hour, or can be delayed. However, these scales are not well suited to gynecological emergencies [5], in which the main challenge consists in identifying patients with PLTEs, whose condition may not be immediately alarming but may deteriorate rapidly [3]. Examples of these PLTEs, presenting with acute pelvic pain as a common signal precursor, include ectopic pregnancy [3,6], adnexal torsion [7] or tuboovarian abscess [8] which can lead to hemodynamic instability, organ failures, severe morbidity and death. Triage tools specifically designed for gynecological emergencies may be useful to rapidly identify patients in whom endovaginal ultrasonography by a gynecologist or radiologist may detect a condition requiring prompt treatment, thus protecting the patient from life-threatening or function-threatening events [6,8,9].

A self-assessment questionnaire for gynecological emergencies (SAQ-GE) recently developed by our group for the assessment of acute pelvic pain in women with gynecologic emergencies has been used to build clinical prediction rules for tubal rupture complicating ectopic pregnancy [10] and for adnexal torsion [11]. Our objective here was to develop and validate a clinical prediction rules for identifying PLTEs in emergency room patients with acute pelvic pain, based on SAQ-GE items.

## Methods

### Ethical aspects

The study was approved by the French Department of Higher Education and Research (n°06.336) and by the French National Committee for Information Technology and Individual Liberties (n°906253).

### Study design and setting

We conducted a prospective multicenter study in five gynecology departments in the Paris metropolitan area, France. Four departments were in teaching hospitals (Poissy-Saint Germain en Laye, Créteil, Port-Royal, and Louis Mourier) and one was in a general hospital (Versailles).

### Selection of Participants

From September 2006 to April 2008, all patients at least 18 years of age who presented to study-center gynecological emergency rooms with acute pelvic pain were eligible to complete the SAQ-GE on a voluntary basis. Exclusion criteria were a history of chronic pelvic pain, neurological or psychiatric disease, hemodynamic instability, and no knowledge of French. Patients with a verbal 11-point numerical rating scale (NRS) pain score lower than 1 and those with bartholinitis or breast pain were excluded.

### Self-Assessment Questionnaire for Gynecological Emergencies (SAQ-GE)

The SAQ-GE was developed using a qualitative method [12] and advice from a panel of French experts, as reported in detail elsewhere [10,11]. The 89 items cover six domains: (i) qualitative description of pain, (ii) intensity of pain, (iii) location and (iv) time-course of pain, (v) vaginal bleeding, and (vi) other signs.

The SAQ-GE was completed by the patients after appropriate initial pain management and before diagnostic investigations or surgery. The nurses collected the completed questionnaires, which were not made available to the physicians. Thus, in this non-interventional study, all diagnostic and therapeutic decisions were made without knowledge of the questionnaire replies.

### Methods and measurements

The final diagnosis was the diagnosis at hospital discharge established based on the physical examination, abdominal and endovaginal ultrasound, routine biology (if needed), computed tomography (CT) of the abdomen and pelvis (if needed), and surgical procedures (if needed: laparoscopy, dilatation and curettage, or diagnostic hysteroscopy). The diagnosis of ectopic pregnancy was based on laparoscopy or on an algorithm [13,14], with laparoscopy being performed when a complication was suspected (i.e., abundant hemoperitoneum with active bleeding and/or tubal rupture), as well as in patients with contraindications to medical treatment. Pelvic inflammatory disease was diagnosed based either on laparoscopy, if deemed necessary, or on noninvasive diagnostic models [15,16]. Other diagnoses based on surgical findings were abundant hemoperitoneum related to ovarian cyst rupture, adnexal torsion, appendicitis, and intestinal obstruction.

Among patients who did not undergo emergency laparoscopy, those who were pregnant were followed until a definitive diagnostic was made [17]. In nonpregnant patients, when the findings of all examinations were deemed normal and the pain subsided with appropriate analgesia by the end of the visit or hospitalization, a diagnosis of idiopathic acute pelvic pain was made. After discharge, patients were encouraged to return to the gynecological emergency room in the event of pain recurrence.

### Outcomes

For the purpose of the study, patients were classified according to whether they had a prospectively recorded diagnosis of PLTE. PLTEs were defined as gynecological or nongynecological disorders causing acute pain and associated with a high risk of complications likely to cause residual impairments, severe morbidity, or death within a short period in the absence of appropriate emergency surgical or radiological treatment [3]. This definition included (i) ectopic pregnancy with tubal rupture or active bleeding or fetal cardiac activity or hemoperitoneum exceeding 300 mL [9,18]; (ii) complicated pelvic inflammatory disease with tuboovarian abscess or pelvic peritonitis [8,15,19]; (iii) adnexal torsion [11]; (iv) hemoperitoneum exceeding 300 mL due to rupture of hemorrhagic ovarian cysts or other gynecological causes (uterine rupture in the first trimester of pregnancy, rupture of a pedunculated uterine fibroid, rupture of an arteriovenous malformation, or uterine perforation); (v) appendicitis; and (vi) intestinal obstruction.

### Analysis

We randomly assessed two-thirds of the patients to the derivation dataset and the remaining third to the validation dataset. All statistical tests were done using Stata 11.0 (Stata Corp., College Station, TX, USA).

SAQ-GE replies of patients with a final diagnosis of PLTE were compared to those of the other patients by univariate analysis using Pearson’s chi-square test or Fisher’s exact test. Variables significantly associated with PLTE with *P* values <0.05 were classified as possible predictors. For each of these variables, we computed sensitivity, specificity, the positive likelihood ratio (Lr+) and negative likelihood ratio (Lr-), and the crude diagnostic odds ratio with their 95% confidence interval (95% CI).

Variables significantly associated with PLTEs by univariate analysis were used for multivariable analysis by recursive partitioning to create a decision tree based on the best combination of variables. The decision tree identified groups at high, intermediate, and low risk for PLTEs based on the sequential Lr values [20]. When a data was missing for a patient, it was considered absent. For each of the three groups, we computed the probability of PLTE with the 95% CI. Sensitivity of the decision tree was defined as the number of patients with PLTEs in the high- and intermediate-risk groups over the total number of patients with PLTEs.

Finally, we assessed the performance of the decision tree in the validation dataset.

## Results

### Characteristics of the study patients

At the five study centers, 574 of about 992 eligible patients completed the SAQ-GE. Among them, 516 met our inclusion criteria and were entered into the study. A final diagnosis of PLTE was made in 145 (28.1%) patients. Table 1 lists the main patient characteristics and diagnoses in the overall population of 516 patients, of whom 344 were randomly allocated to the derivation dataset and 172 to the validation dataset. PLTEs were diagnosed in 96 (27.9%) derivation-dataset patients and 49 (28.5%) validation-dataset patients. Patient characteristics were not significantly different in the two datasets (data not shown).

**Table 1:** Characteristics and main diagnoses in the study patients

	**Overall population N = 516**	**PLTE N = 145**	**Other N = 371**
Age in years, mean ± SD	31.6 ± 7.7	30.7 ± 7.9	31.9 ± 7.6
Gravidity, median [range]	2 [0–11]	2 [0–9]	2 [0–11]
Parity, median [range]	1 [0–7]	1 [0–4]	1 [0–7]
Contraception, n/N (%)	136/504 (27.0)	40/141 (28.4)	96/363 (26.5)
NRS pain score at admission, mean ± SD	6.4 ± 2.7	6.8 ± 2.7	6.2 ± 2.7*
**Diagnosis**			
Ectopic pregnancy, n (%)	148 (28.7)	77 (53.1)	71 (19.1)
Pelvic inflammatory disease, n (%)	73 (14.1)	25 (17.2)	48 (12.9)
Uncomplicated ovarian cyst, n (%)	70 (13.6)	NA	70 (18.9)
Adnexal torsion, n (%)	31 (6.0)	31 (21.4)	NA
Appendicitis, n (%)	6 (1.2)	6 (4.1)	NA
Ruptured cyst with hemoperitoneum > 300 mL, n (%)	5 (1.0)	5 (3.5)	NA
Miscarriage, n (%)	79 (15.3)	NA	79 (21.3)
Myoma necrobiosis, n (%)	15 (2.9)	NA	15 (4.0)
Urologic disease, n (%)	10 (1.9)	NA	10 (2.7)
Ovarian hyperstimulation, n (%)	7 (1.4)	NA	7 (1.9)
Other diagnosis, n (%)	72 (13.9)	1 (0.7)‡	71 (19.1)

PLTE, potentially life-threatening emergencies; NRS, numerical rating scale for pain severity; NA, not applicable; SD, standard deviation.

*P < 0.05, Student’s t test; ‡Intestinal obstruction.

### Main results

**Figure 1:**
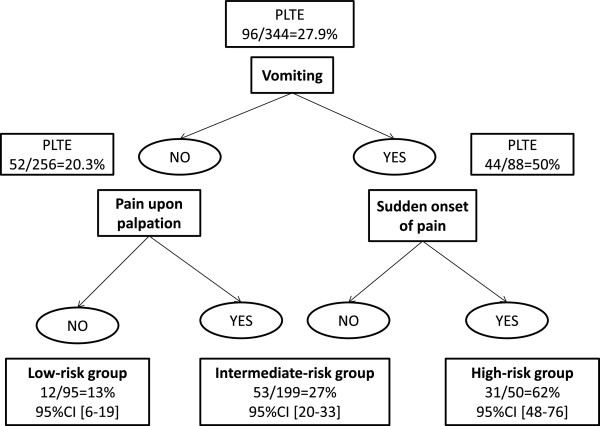
Decision tree for classifying the risk of potentially-life-threatening emergency in patients presenting to gynecological emergency rooms with acute pelvic pain.

Table 2 reports the results of the univariate analysis. None of the SAQ-GE items had Lr + values greater than 4 or Lr- values lower than 0.25.Figure 1 shows the decision tree, in which three items are taken into account sequentially: vomiting, sudden onset of pain, and pain upon self-palpation. Patients with no vomiting or pain upon palpation are at low risk, with a probability of PLTE of 13% (95% CI, 6%-19%). The intermediate risk group is defined based on either no vomiting but pain upon self-palpation or vomiting but no sudden onset of pain; the probability of a PLTE is 27% (95% CI, 20%-33%). In the high-risk group, with both vomiting and sudden-onset pain, the probability of a PLTE is 62% (95% CI, 48%-76%), ruling out PLTE with a specificity of 92.3%; (95% CI, 89%-96%) (Figure 1). Sensitivity of the decision tree was 87.5% (95% CI, 81%-94%).

**Table 2:** **SAQ-GE items significantly associated (*P*** **< 0.05) with PLTE by univariate analysis in the derivation dataset**

	Total, n/N* (%)	**PLTE, n/N (%)**	**Other, n/N (%)**	**Se (%)**	**Sp (%)**	**LR+**	**LR-**	**DOR [95% CI]**
**Prior surgery for ovarian cyst**	53/338 (15.6)	23/93 (24.7)	30/245 (12.2)	24.7	87.8	2.0	0.86	2.4 [1.3-4.4]
**No history of pain of similar intensity**	175/336 (52.1)	65/95 (58.4)	110/241 (45.6)	58.4	54.4	1.3	0.76	2.6 [1.5-4.3]
**Pain on one side**	184/337 (54.6)	69/92 (75.0)	115/245 (46.9)	75.0	53.1	1.6	0.47	3.4 [2.0-5.9]
**Ovarian pain**	210/337 (62.3)	69/92 (75.0)	141/245 (57.6)	75.0	42.4	1.3	0.59	2.2 [1.3-3.8]
**Pain radiating to the stomach**	59/336 (17.6)	23/93 (24.7)	36/243 (14.8)	24.7	85.2	1.7	0.88	1.9 [1.0-3.4]
**Sudden onset of pain**	170/333 (51.0)	61/94 (64.9)	109/239 (45.6)	64.9	54.4	1.4	0,64	2.2 [1.3-3.6]
**Pain exacerbated by movements**	248/337 (73.6)	81/94 (86.2)	167/243 (68.7)	86.2	31.3	1.3	0.44	2.8 [1.5-5.5]
**Pain upon self-palpation**	222/335 (66.3)	75/91 (82.4)	147/244 (60.3)	82.4	39.7	1.4	0.44	3.1 [1.7-5.7]
**Vomiting**	88/338 (26.0)	44/94 (46.8)	44/244 (18.0)	46.8	82.0	2.6	0.65	4.0 [2.3-6.9]
**Radiating pain**	35/309 (11.3)	19/87 (21.8)	16/222 (16.2)	21.8	83.8	1.3	0.93	3.6 [1.7-7.5]
**Penetrating pain**	114/329 (34.6)	44/92 (47.8)	70/237 (29.5)	47.8	70.5	1.6	0.74	2.2 [1.3-3.6]
**Twisting pain**	72/329 (21.9)	34/93 (36.6)	38/236 (16.1)	36.6	83.9	2.3	0.76	3.0 [1.7-5.3]
**Pain leading to syncope**	25/332 (7.5)	12/94 (12.8)	13/238 (5.5)	12.8	94.5	2.3	0.92	2.5 [1.1-5.8]
**Pain with sensation of oppression**	82/333 (24.6)	34/94 (36.2)	48/239 (20.1)	36.2	79.9	1.8	0.80	2.3 [1.3-3.8]
**Torturous pain**	68/333 (20.4	29/94 (30.8)	39/239 (16.3)	30.8	83.7	1.9	0.83	2.3 [1.3-4.0]

*Because of missing data, the total may be different from 344.

PLTE, potentially life-threatening emergency; Se, sensitivity; Sp, specificity; LR, likelihood ratio; DOR, diagnostic odds ratio; 95% CI, 95% confidence interval.

In the validation dataset, the diagnostic performance characteristics of our decision tree were similar to those in the derivation dataset, with most of the validation-dataset values being within the 95% CI for the derivation-dataset values. The PLTE probability was 16.3% in the low-risk group, 30.6% in the intermediate-risk group, and 44% in the high-risk group, ruling out the diagnosis of PLTE with a specificity of 88.6%. Sensitivity of the decision tree was 83.7% in the validation dataset.

## Discussion

We built a decision tree for triaging women presenting to the emergency room with acute pelvic pain using a standardized yes/no items from a self-questionnaire. The decision tree relies on three simple items: vomiting, pain upon self-palpation, and sudden onset of pain. It separates three groups of patients, at low, intermediate, and high risk for PLTEs, respectively. Sensitivity of the decision tree was 87.5% (95% CI, 81%-94%).

The time to management of gynecological emergencies is the sum of four periods: time from symptom onset to arrival; time from arrival to the first medical assessment; time from the first medical assessment to the diagnosis, which usually required pelvic and endovaginal ultrasonography by a specialist [21]; (iv) and time from the diagnosis to the implementation of specific treatment, if any is needed. Our decision tree may diminish the time from arrival to the first medical assessment by helping the nurses to identify patients with suspected PLTEs. In a previous study, mean time from arrival to ultrasonography was 84 minutes in a gynecological emergency room, and far longer times were found in general emergency rooms [2]. Then, this decision tree can speed up the use of ultrasound examination that has proven to be reliable for the diagnosis of surgical emergencies [22].

Most triage tools use clinical decision rules that separate patients into five triage categories depending on the acceptable time to medical management [4,23]. These rules are usually established by consensus among experts, both for the triage category and for the acceptable time to medical management [23]. We used a different approach, using statistical data to separate the patient groups and focusing on the diagnosis rather than on acceptable time to management. Our classification system could serve as a reference for classifying gynecological emergencies. Our next step will be to determine the acceptable time to medical management in each of the three groups, before validating the decision tree in other settings and evaluating its impact in clinical practice [23]. Moreover, our triage tool is not expensive. Then, it could be used, after scaling up, in developing countries where institutional and human resources are often low, in order to decrease women’s severe morbidity.

A rigorous statistical approach was used to develop our decision tree, in contrast to the methods generally used by consensus panels [23]. Decision trees developed using recursive partitioning are simple to use. No computations are needed to determine the risk group to which a given patient belongs. In addition, recursive partitioning has been proven equivalent to logistic regression in terms of diagnostic efficiency [24,25]. We also found that recursive partitioning and logistic regression performed similarly in our datasets (data not shown).

The high predictive values of our model may seem surprising in the light of pathophysiological considerations. Our definition of PLTE encompassed a variety of conditions that differ regarding the pathophysiological mechanisms responsible for pain [26,27]. However, when we built the SAQ-GE, our main hypothesis was that words used by women to describe acute pelvic pain and concomitant symptoms reflect an underlying sensory experience shared by various pathological and anatomical abnormalities [27] and not the symptoms of a specific disease. For instance, vomiting strongly predicted both tubal rupture [10] and adnexal torsion [28]. Most gynecological emergencies may involve the same general protective mechanisms triggered in response to danger, such as activation of the autonomic nervous system [26,27]. Thus, acute pelvic pain and other symptoms as described by women may serve as warning signals that can provide diagnostic orientation.

## Limitations

One limitation of our study is related to our definition of PLTE. This definition was not established by consensus among a panel of experts [29]. Nevertheless, our definition of PLTE is consistent with clinical reality in patients with gynecological emergencies. For instance, ectopic pregnancy can be life threatening in the event of tubal rupture with hemodynamic shock from massive intraabdominal bleeding. In this situation, substandard care is often related to misdiagnosis [3,6]. We extended this concept to all gynecological emergencies that may not pose an immediate threat but may worsen rapidly. We used acute pelvic pain as the warning signal for such situations. Our definition of PLTE is similar to that used pragmatically in general emergency rooms with the goal of identifying conditions likely to cause serious subsequent manifestations (http://www.acem.org.au/media/policies_and_guidelines/G24_Implementation__ATS.pdf). In patients with PLTEs as defined for our study, an earlier and more accurate diagnosis allows the rapid provision of appropriate care, thereby improving patient outcomes in terms of both morbidity and mortality.

Another limitation may be overfitting of the decision tree to our data. However, the validation study in the third of our population not used to build the decision tree showed similar diagnostic performance characteristics and substantial overfitting was also prevented by constructing the SAQ-GE in a preliminary study involving different patients and experts.

## Conclusion

In summary, our decision tree is the first dedicated to the diagnosis of PLTEs with a 87.5% sensitivity. In addition, it relies on only three simple items of a self-questionnaire. We plan to study the extent to which our decision tree decreases time to appropriate management and improves outcomes in patients presenting with acute pelvic pain to crowded emergency rooms.

## Competing interest

The authors declare that they have no competing interests.

## Authors' contributions

CH and AF wrote the manuscript. AF, AD and BF designed the study. AAC, CH and AF collected the datas. CH, AD and AF performed the statistical analysis.
